# *Manglietia pubipedunculata* (Magnoliaceae), a new species from Yunnan, China

**DOI:** 10.1371/journal.pone.0210254

**Published:** 2019-03-13

**Authors:** Xiao-Min Hu, Qing-Wen Zeng, Ya-Si Liu, Lin Fu, Ru-Chun Xi, Hong-Feng Chen, Xiao-Mei Deng

**Affiliations:** 1 Guangdong Key Laboratory for Innovative Development and Utilization of Forest Plant Germplasm, College of Forestry and Landscape Architecture, South China Agricultural University, Guangzhou, Guangdong, People’s Republic of China; 2 Key Laboratory of Plant Resources Conservation and Sustainable Utilization, South China Botanical Garden, Chinese Academy of Sciences, Guangzhou, Guangdong, People’s Republic of China; 3 State Key Laboratory for Conservation and Utilization of Subtropical Agro-bioresources, Guangzhou, Guangdong, People’s Republic of China; National University Comahue, ARGENTINA

## Abstract

A new species, *Manglietia pubipedunculata* Q. W. Zeng & X. M. Hu (Magnoliaceae) is described and illustrated from Yunnan, China. In addition to macromorphological examination, we comparatively studied on micromorphology of leaf epidermis, leaf structure, and epidermal cell on the sclerotesta. This new species is similar to *M*. *kwangtungensis* in terms of having dense pubescence, however, their pubescence are quite different. *Manglietia pubipedunculata* has appressed, compressed, shorter and sparser pubescence consisting of single or two cells. Moreover, it differs from *M*. *kwangtungensis* by showing shorter and thicker peduncles, longer styles, basal carpels covered with sparsely brown appressed pubescence, and more ovules per carpel. Furthermore, the new species has thinner leaves, brown and rugged surfaces on sclerotesta, and the alveolate cell pattern consisting of pentagon or hexagon cells with papilla on secondary cell wall under the observation by SEM. The phylogenetic analysis from two nuclear PHYA and LEAFY and chloroplast *trn*H-*psb*A sequences of 11 taxa reveals that *M*. *pubipedunculata* is a distinct species.

## Introduction

China is one of the countries with the highest number of species in the Magnoliaceae throughout the world. More than 100 species of Magnoliaceae are found in China [1]. Southwest and South China including Yunnan, Guangxi, Guangdong, Hainan, Guizhou and the neighbouring areas are considered to be the center of modern distribution and diversity of Magnoliaceae in the world [1–4].

The genus *Manglietia* was proposed by Blume [5], but its systematic status has been long debated by taxonomists, some suggested that *Manglietia* should be reduced to *Magnolia* [6–16], while others insisted *Manglietia* should be a separate genus based on the number of ovules per carpel [17], the characteristics of leaf epidermis [18], foliar sclereids [19], structure of leaves [20], *ndh*F and *mat*K sequence [21–25], the morphology of seed endotesta at chalazal region [26], sclerotesta [27] and pollen [28, 29]. Through long-term thorough research on Magnoliaceae, Liu proposed a system and treated *Manglietia* as a separate genus [30]. This treatment was accepted widely in local floras and Flora of China [1, 31–35], as well as revising the family Magnoliaceae [36–42]. More recently, Xia et al. [43] proposed another different taxonomic system of Magnoliaceae, in which the genus *Manglietia* was also treated as a separate genus. In this paper, we followed this treatment.

During the field survey in Yunnan Province, we found a species belonging to *Manglietia*, which is called “Maotao” by the local people. The species was thought to be *M*. *kwangtungensis* at first glance, but studies on both macromorphological and micromorphological characters of leaf epidermis, leaf structure and sclerotesta indicated that it is quite different from *M*. *kwangtungensis*. Therefore, it is described and illustrated here as a new species.

## Materials and methods

### Ethics statement

Plants studied in this work were collected in Donggualin, Daxinzhai Village, Miechang Town, Maguan County, Wenshan Prefecture, Yunnan Province, and South China Botanical Garden, Chinese Academy of Sciences, Guangzhou City, Guangdong Province, and Nankun Moutain Nature Reserve, Yonghan Town, Longmen County, Huizhou City, Guangdong Province. The first location referred was not protected in any way. Permissions to enter and collect samples in the second and third location referred above was issued by Magnolia Garden, South China Botanical Garden, Chinese Academy of Sciences, and Management Office of Nankun Moutain Nature Reserve.

### Morphological observation

Morphology of the species was examined and compared to that of *M*. *kwangtungensis* based on both freshly collected samples in the field, and the herbarium specimens from PE, IBSC, KUN, SYS and GF, as well as the information gathered in the literature. The herbarium acronyms follow the Index Herbarium [44].

### Leaf epidermis and structure

Small pieces (1 cm × 0.5 cm) near the midrib of fully developed leaves of *M*. *pubipedunculata* and *M*. *kwangtungensis* were taken, extracted and fixed in 0.25% of glutaraldehyde solution for more than 12 hours. In order to examine leaf structure, the samples were cut into smaller pieces (0.5 cm × 0.1 cm) by the new sharp blade, washed three times with 0.1 mol phosphate buffer for 2 hours, dehydrated with a graded series of ethanol (30%, 50%, 70%, 80%, 90%) for 15 minutes, respectively, then treated three times with 100% of alcohol and tert-butyl alcohol for 10 minutes. After these treatments, the samples were frozen, then dried with vacuum dryer. The abaxial and adaxial surfaces, and transverse section of leaves were mounted on the surface of brass stubs with double-sided tape, respectively, and coated with palladium gold using a SPI-MODLE sputter coater. Characters were observed under the JEOL JSM-6360 LV scanning electron microscope operating at 25 kv, and were measured by imaging analyzer (Smile View 2.1; JEOL Tokyo, Japan). Voucher specimens of both plants from which the leaves originated (Q. W. Zeng & X. M. Hu 00240, X. M. Hu 00311) were placed in IBSC. The terminology follows Pant et al. [45], Baranova [18], and Cai & Hu [20].

### Sclerotesta morphology

The fresh mature seeds were soaked in warm water with a little washing powder for two days, cleaned with hands to remove the exotesta and mesotesta, and dried naturally, then observed, measured and photographed directly under SV11 ZEISS stereomicroscope. Voucher specimens of both plants from which the seeds originated (Q. W. Zeng & X. M. Hu 00240, Q. W. Zeng & X. M. Hu 00256) were placed in IBSC. The terminology follows Tiffney [46] and Xu [27].

### Sclerotesta epidermal cell

After removing the exotesta and mesotesta, the fresh mature seeds were put in a solution of 1:1 xylene and acetone, cooked continuously under 70 °C thermostat metal bath (JS-400A) for one week, and washed at least 3 times with a new solution of 1:1 xylene and acetone in the ultrasonic cleaning machine (1510E-MT) for 30 minutes. Then, the seeds were cleaned with a small amount of 100% of ethanol and aired in a fume hood. Finally, the seeds were mounted on the surface of brass stubs with double-sided tape, and coated with palladium gold using a SPI-MODLE sputter coater. Characters were observed under the JEOL JSM-6360 LV scanning electron microscope operating at 25 kv, and were measured by imaging analyzer (Smile View 2.1; JEOL Tokyo, Japan). Voucher specimens of both plants from which the seeds originated (Q. W. Zeng & X. M. Hu 00240, Q. W. Zeng & X. M. Hu 00256) were placed in IBSC. The terminology follows Karcz et al. [47].

We sequenced three samples of *M*. *pubipedunculata* and two samples of *M*. *kwangtungensis* for this work. In addition, we downloaded sequences for 9 species of Magnoliaceae from GenBank. GenBank accession numbers for all the DNA sequences and voucher information are given in S1 Table.

### Molecular markers

Total cellular DNA was isolated from silica-dried plant leaves using the modified CTAB method [48]. Two nuclear genes (PHYA and LEAFY) and chloroplast gene (*trn*H-*psb*A) were analyzed. The primers for PHYA (PHYA-1) and LEAFY (LFY-3) (S2 Table) were modified from those used in Nie et al. [49] based on the sequences of *M*. *moto* (EU 849973.1) and *M*. *insignis* (EU849837.1), respectively. The *trn*H-*psb*A gene region was amplified using the primers as described by Sang et al. [50]. PCR product was sequenced by DNA Analyzer (Applied Biosystems 3730xl).

### Sequence alignment and phylogenetic analysis

We aligned the sequences using Clustal W [51], followed by manual adjustments in Bioedit [52]. Test of substitution saturation showed sequences can be used to build phylogenetic tree for Iss.c (0.7971) > Iss (0.0292), and Prob (Two-tailed) was 0.000. Test for homoplasy using PAUP * version 4.0a [53] showed three genes in this study can be combined to analysis (P = 0.1>0.05). Phylogenetic analyses were carried out using Maximum Parimony (MP) and Maximum Likelihood (ML) as implemented in PAUP* version 4.0 [53] and RAxML-7.0.3 [54]. The MP analysis was performed using heuristic searches with 1000 bootstrap (BS) replicates, the ML analysis used the default GTRCAT_GAMMA nucleotide substitution model. Bootstrap support for ML topologies was inferred using the fast bootstrap algorithm with 1000 replicates. *Michelia cavaleriei* was used as outgroup because it belongs to Magnoliaceae, but was not the species of *Manglietia*.

### Nomenclature

The electronic version of this article in Portable Document Format (PDF) in a work with ISSN or ISBN will represent a published work according to the International Code of Nomenclature of algae, fungi, and plants, and hence the new names contained in the electronic publication of a PLOS ONE article are effectively published under that Code from the electronic edition alone, so there is no longer any need to provide printed copies.

In addition, new names contained in this work have been submitted to IPNI, from where they will be made available to the Global Names Index. The IPNI LSIDs can be resolved and the associated information viewed through any standard web browser by appending the LSID contained in this publication to the prefix http://ipni.org/. The online version of this work is archived and available from the following digital repositories: PubMed Central, LOCKSS.

## Results

### Taxonomic treatment

***Manglietia pubipedunculata*** Q. W. Zeng & X. M. Hu, sp. nov.

**Fig 1:**
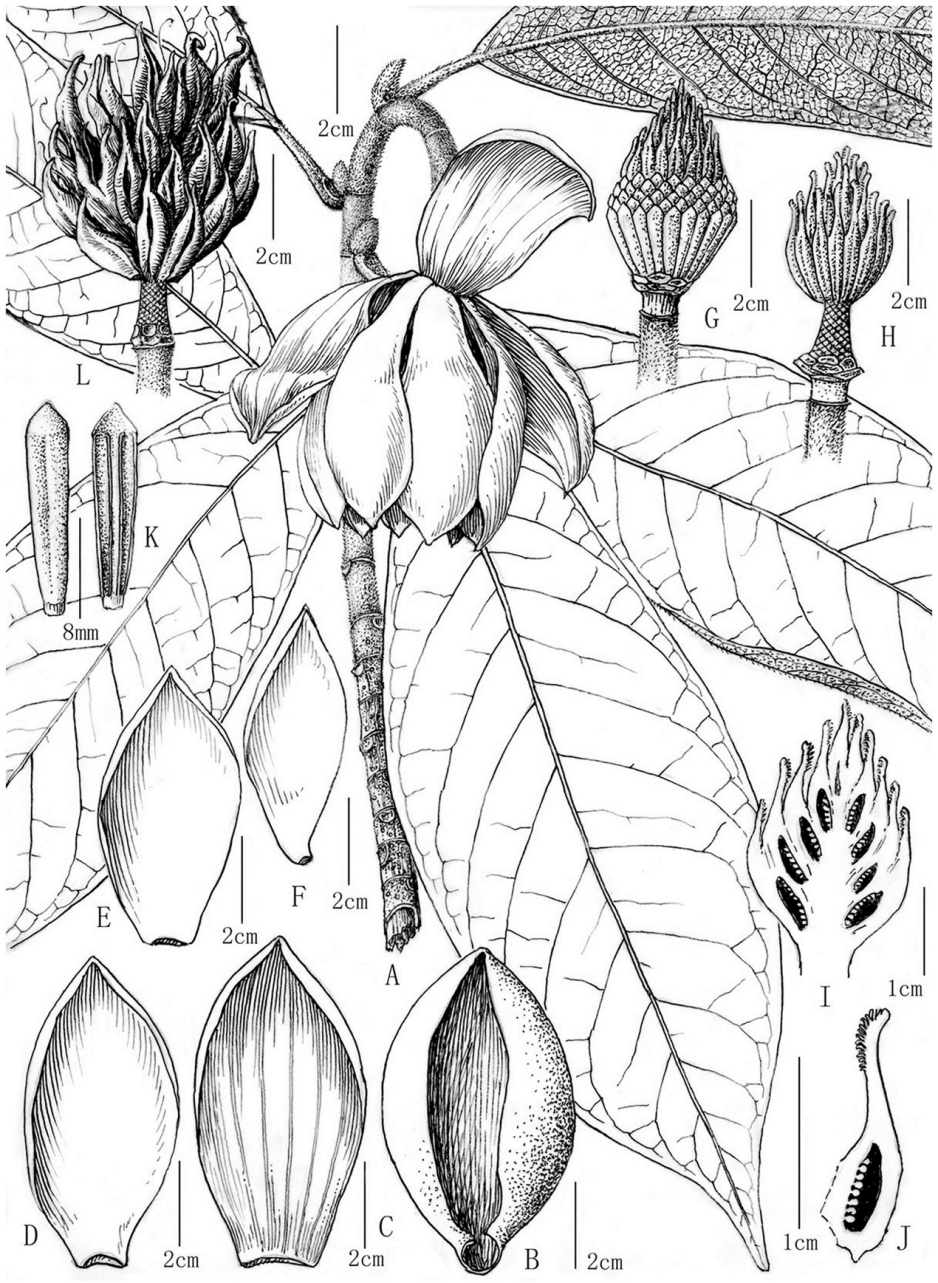
*Manglietia pubipedunculata*. A, flower branch; B, flower bract; C, outer tepal; D, mid tepal; E-F, inner tepal; G, gynoecium with stamens; H, gynoecium; I, longitudinal section of gynoecium; J, longitudinal section of carpel; K, stamens; L, fruit aggregate.

**Fig 2:**
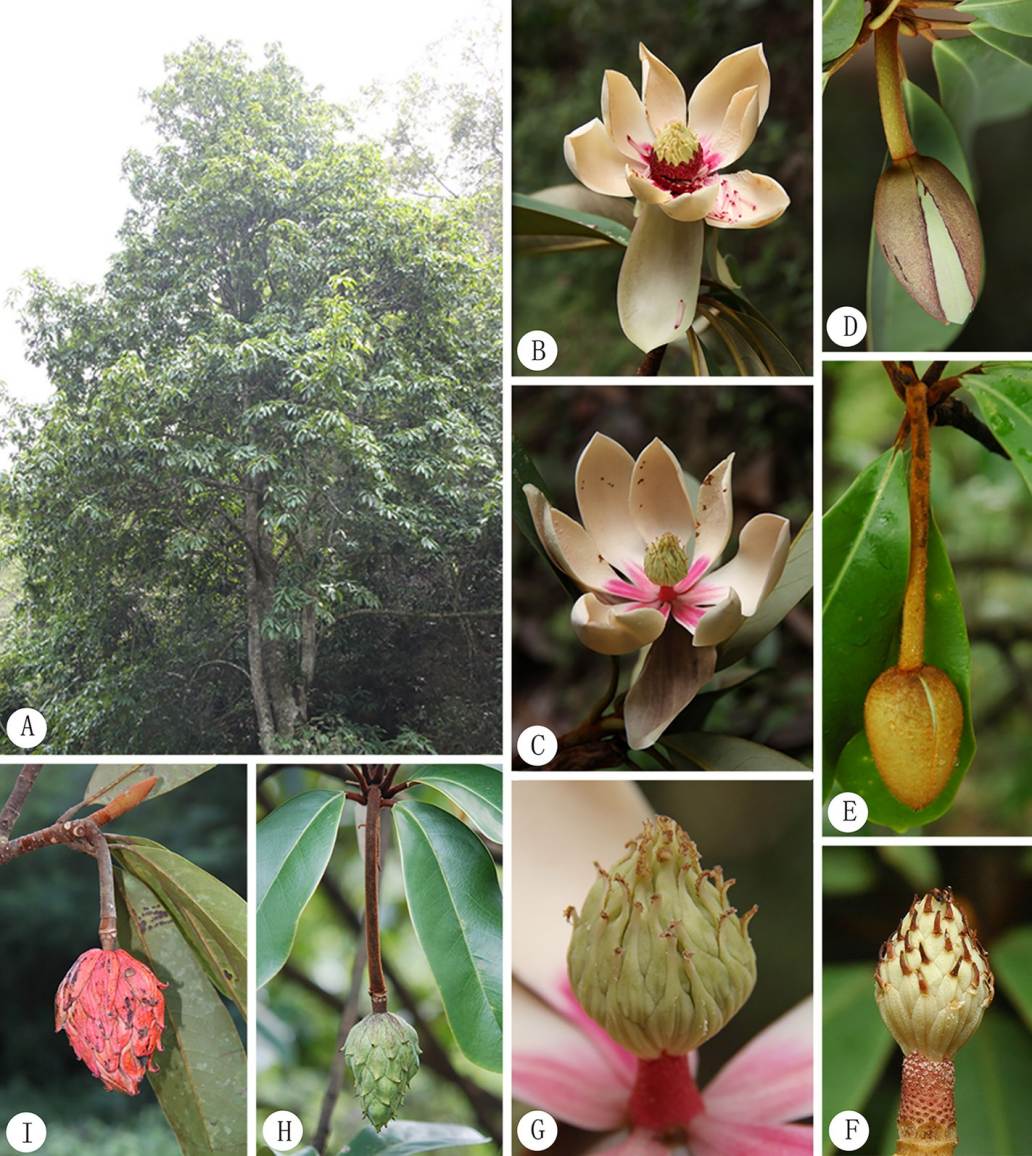
Images of living plants of *Manglietia pubipedunculata* and *M*. *kwangtungensis*. A-D, G, I, *M*. *pubipedunculata*; E, F, H, *M*. *kwangtungensis*. A, habit; B-C, flower; D-E, flower bud; F-G, gynoecium; H-I, fruit aggregate.

[urn:lsid:ipni.org:names: XXXXXXXX-X] (Figs 1, 2A–2D, 2G and 2I).

**Type**. CHINA. Yunnan Province, Wenshan Prefecture, Maguan County, Miechang Town, Daxinzhai Village, Donggualin, Huashikeng, evergreen broad-leaved forests, alt. 1453 m, 104°05'21"E, 22°54'50"N, 14 May 2004, Q. W. Zeng 89 (holotype: IBSC). The same locality, 9 September 2003, Q. W. Zeng 80 (paratype: IBSC).

#### Diagnosis

Species M. kwangtungensis affinis, a qua ramulis, gemmis, petiolis, foliis subtus pedunculisque dense appresse brunneo-pubescentibus, veins reticulatis inconspicuis, tepalis 10−11, 3 exterioribus 8.8−9.8 cm longis et 3.8−4.8 cm latis, carpellis 39, basi sparse appresse pubescentibus, stylis 7−8 mm, pedunculis gracilibus 5.5−7.5 cm longis et 7−8 mm latis, folliculorum rostris 3–5 mm longis differt.

#### Discription

Evergreen trees, up to 35 m high and 80 cm in diam., bark grayish-white; twigs pale green when young, brown when old; buds, young twigs, peduncles, spathecous bracts and fruiting peduncles brown appressed pubescent. Leaves leathery, obovate-elliptic, 20−35 cm long, 6.5−9.5 cm wide, apex long-acuminate, base cuneate, dark green and glabrous above, pale green and rusty-brown appressed pubescent beneath; midribs impressed above, prominent beneath, lateral veins 14−19 on each side, flat above, prominent beneath, reticulation inconspicuous; stipules brown appressed pubescent, adnate to the petioles; petioles 2.3−3.5 cm long, sulcate, thickened at base, stipule scars 1.1−1.5 cm long. Flowers bisexual, solitary and terminal, slightly fragrant; flower buds ovoid, 4.5−5.5 cm long, 3−3.2 cm in diam.; peduncles brown appressed pubescent, pendent, 5.5−7.5 cm long, 0.7−0.8 cm in diam., with 1 bract scar, pedicels rusty-brown pubescent, 2−5 mm long, 0.7−0.8 cm in diam.; tepals 10−11, outer 3 obovate-oblong, nearly leathery, pale green, 8.8−9.8 cm long, 3.8−4.8 cm wide, inner 2 whorls white, thickly fleshy, mid 3 obovate-spathulate, 8.2−9.2 cm long, 3.2−3.8 cm wide, inner 4−5 spathulate, 7.2−7.5 cm long, 2.5−3 cm wide; stamens ca 205, purplish-red, glabrous, 1.6−1.7 cm long, anthers 1.5−1.6 cm long, introrsely dehiscent, filaments ca. 1 mm long, connectives produced into triangular appendages; gynoecium pale green, ovoid, 2.6−3.2 cm long, 1.9−2.2 cm in diam.; carpels ca. 39, 3.5 cm long, sparsely brown appressed pubescent at base, styles 7−8 mm, ovules (9−)10−13(−14) per carpel. Fruit aggregates ovoid, 5−6 cm long, 4.2−4.5 cm in diam.; follicles with 3−5 mm long beak at apex, dehiscent along dorsal sutures; fruiting peduncles rusty-brown appressed pubescent, 6−8 cm long, 0.7−0.8 cm in diam., fruiting pedicels rusty-brown appressed pubescent, 0.5−0.8 cm long, 0.7−0.8 cm in diam.; seeds 2−4 per follicle, compressed ovoid.

**Phenology**. Flowering from May to June, and fruiting from September to October.

**Distribution and habitat**. *Manglietia pubipedunculata* is so far known only from a single location in Maguan County, the southeast of Yunnan Province. It grows in evergreen broad-leaved forests at 1400−1600 m with *M*. *megaphylla*, *M*. *ovoidea* and *Alnus nepalensis*, etc.

**Additional specimens examined**. China. Yunnan Province, Maguan County, Miechang Town, Daxinzhai Village, Donggualin, Huashikeng, evergreen broad-leaved forests, 7 May 2010, Q. W. Zeng & X. M. Hu 00148, 20 September 2010, Q. W. Zeng & X. M. Hu 00240 (IBSC).

### Micromorphology characteristics of leaf epidermis

**Fig 3:**
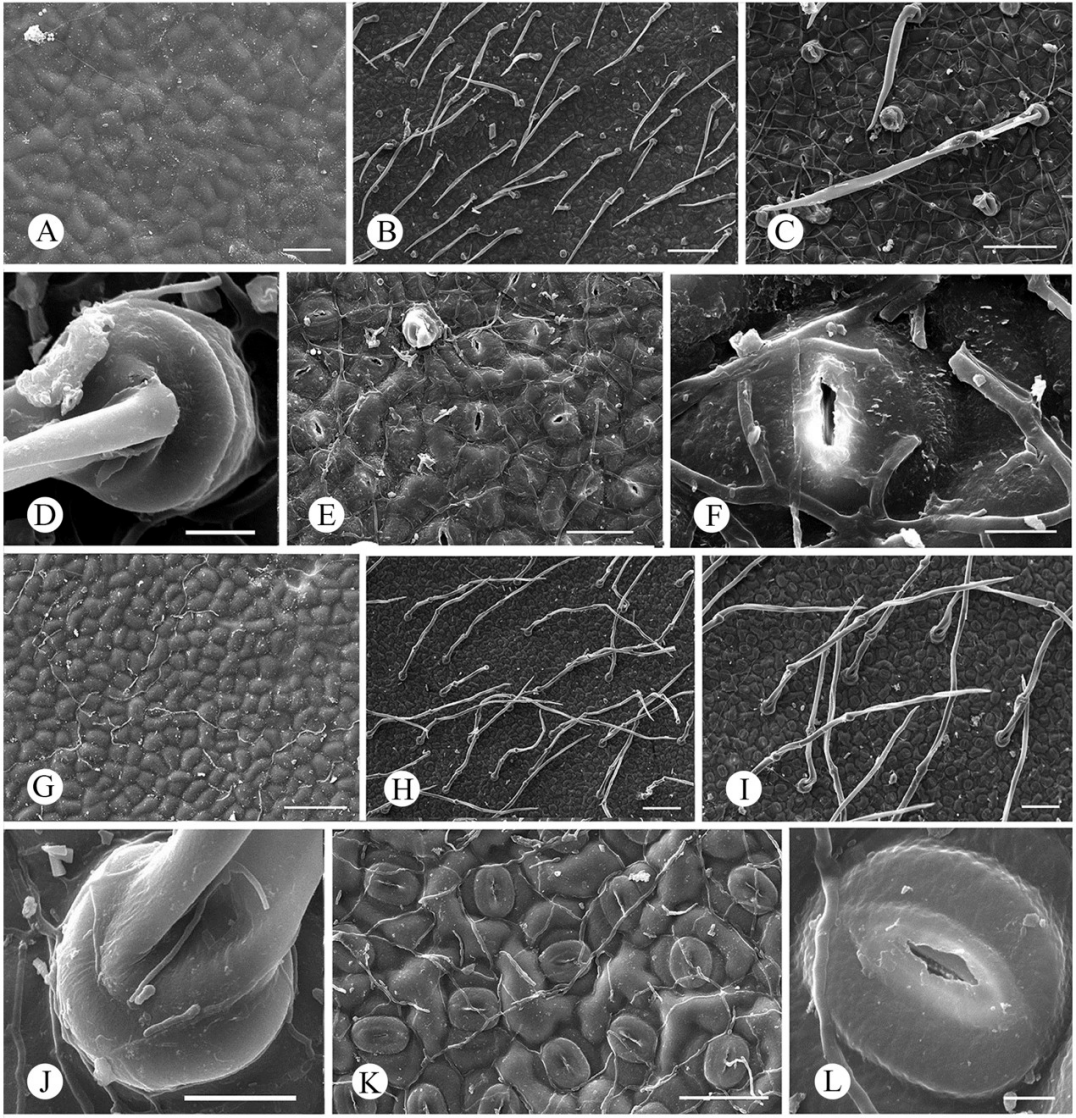
Characteristics of leaf epidermis of *Manglietia pubipedunculata* and *M*. *kwangtungensis*. A-F, *M*. *pubipedunculata*; G-L, *M*. *kwangtungensis*. A, G, outline of upper epidermis; B, C, H, I, pubescence of lower epidermis; D, J, pubescence base of lower epidermis; E, F, K, L, stomata apparatus of lower epidermis. Scale bar: A, E, K = 50 μm; B, H = 200 μm; C, G, I = 100 μm; D, F = 10 μm; J = 20 μm; L = 5 μm.

The adaxial epidermis of two species were glabrous (Fig 3A and 3G), epidermal cells seemed irregularly in outline (Fig 3A and 3G). There were lots of pubescence (Fig 3B, 3C, 3H and 3I) growing on round-table-shape placentas (Fig 3D and 3J) and stomatal apparatus (Fig 3E, 3F, 3K and 3L) exsiting on the abaxial epidermis. For *M*. *pubipedunculata*, the epidermal cells were a bit blurred in outline (Fig 3A), the pubescence was appressed, compressed, and consisted of single or two cells (Fig 3B and 3C), with the average length of 364.2 μm. It covered 40 pubescence per mm^2^. The stomatal apparatus (Fig 3E and 3F) was protrudent slightly and elliptical with the size of 26.9 × 22.4 μm. It had 274 stomas per mm^2^. For *M*. *kwangtungensis*, the pubescence was cocked, spiral, and consisted of three cells (Fig 3H and 3I), with the average length of 813.3 μm. It covered 12 pubescence per mm^2^. The stomatal apparatus (Fig 3K and 3L) was protrudent apparently and elliptical with the size of 29.8 × 22.7 μm. It had 309 stomas per mm^2^.

### Micromorphology characteristics of leaf structure

**Fig 4:**
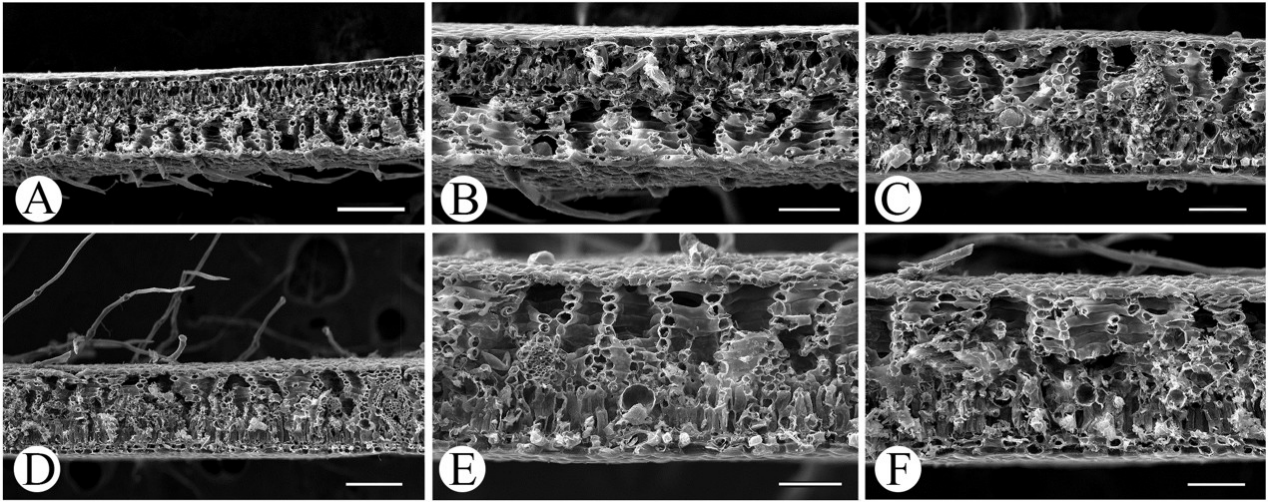
Characteristics of leaf structure of *Manglietia pubipedunculata* and *M*. *kwangtungensis*. A-C, *M*. *pubipedunculata*; D-F, *M*. *kwangtungensis*. Scale bar: A, D = 200 μm; B, C, E, F = 100 μm.

The thickness of leaves were different, the average of *M*. *pubipedunculata* was 217.3 μm, with upper epidermis of 29.1 μm, palisade tissue of 62.6 μm, and spongy tissue of 105.9 μm, while the average of *M*. *kwangtungensis* was 299.7 μm, with upper epidermis of 34.7 μm, palisade tissue of 88.2 μm, and spongy tissue of 153.8 μm. One continuous layer of cells were observed under the upper epidermis (Fig 4A, 4C, 4D and 4F), and oil cells were distributed randomly in mesophyll in these two species (Fig 4B–4F).

### Sclerotesta morphology

**Fig 5:**
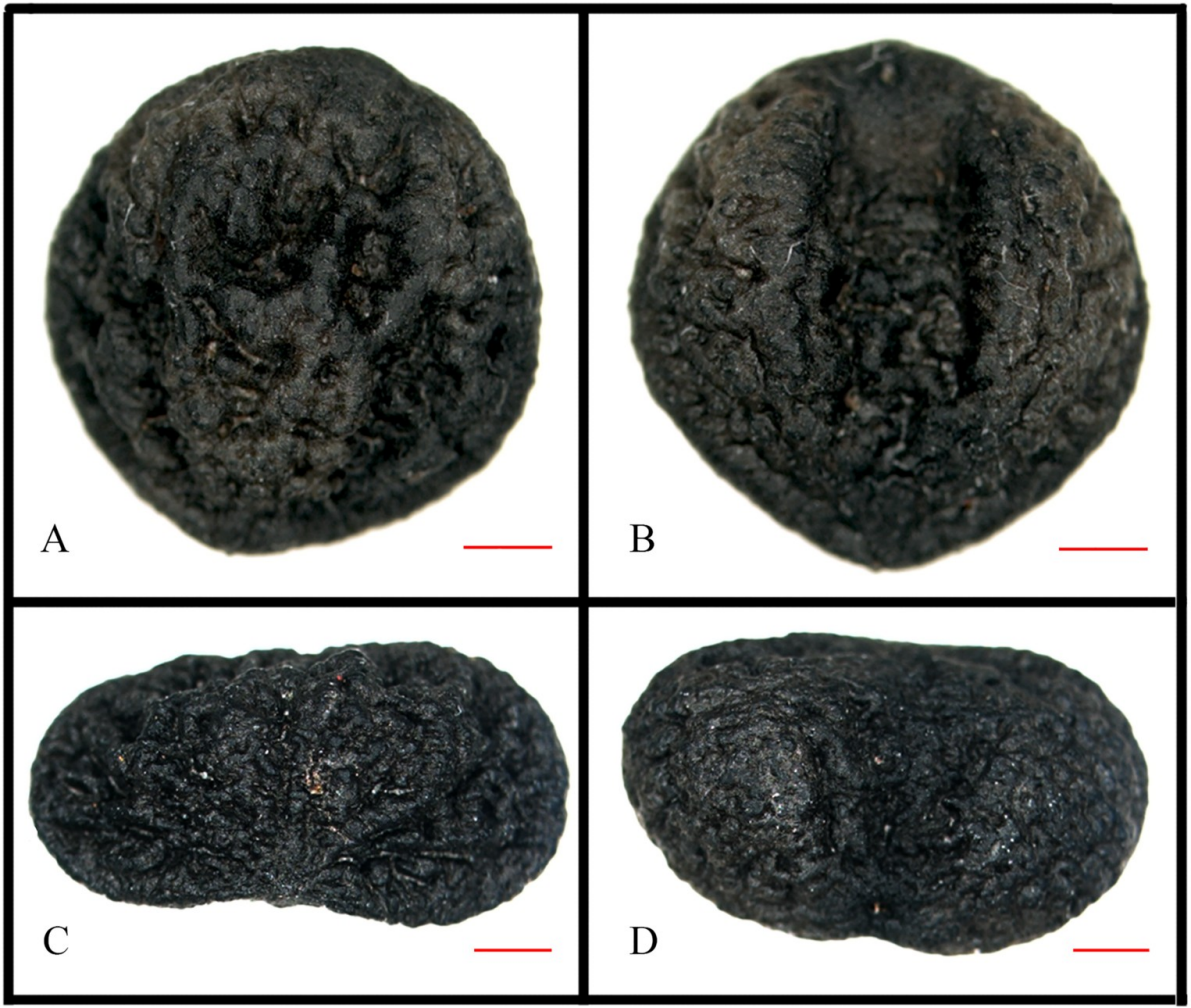
Sclerotesta morphology of *Manglietia pubipedunculata* and *M*. *kwangtungensis* under stereomicroscope. A, B, *M*. *pubipedunculata*; C, D, *M*. *kwangtungensis*; A, C, dorsal surface of seeds; B, D, ventral surface of seeds. Scale bar: 1 mm.

Under the stereomicroscope, the chalazal of the two species was pore, which was round and occurred on the upper ventral face (Fig 5B and 5D). The colour of *M*. *pubipedunculata* was brown, the shape was oblong in outline (Fig 5A), the size was 6.09 × 5.9 mm. The raphal sinus was broad and deep (Fig 5B). The sculpture of surface was rugged (Fig 5A and 5B). However, the colour of *M*. *kwangtungensis* was black, the shape was bean-like or cordate in outline (Fig 5C and 5D), the size was 4.07 × 6.54 mm. The raphal sinus was shallow (Fig 5D). The sculpture of surface was corrugated (Fig 5C and 5D).

### Characteristics of sclerotesta epidermal cell

**Fig 6:**
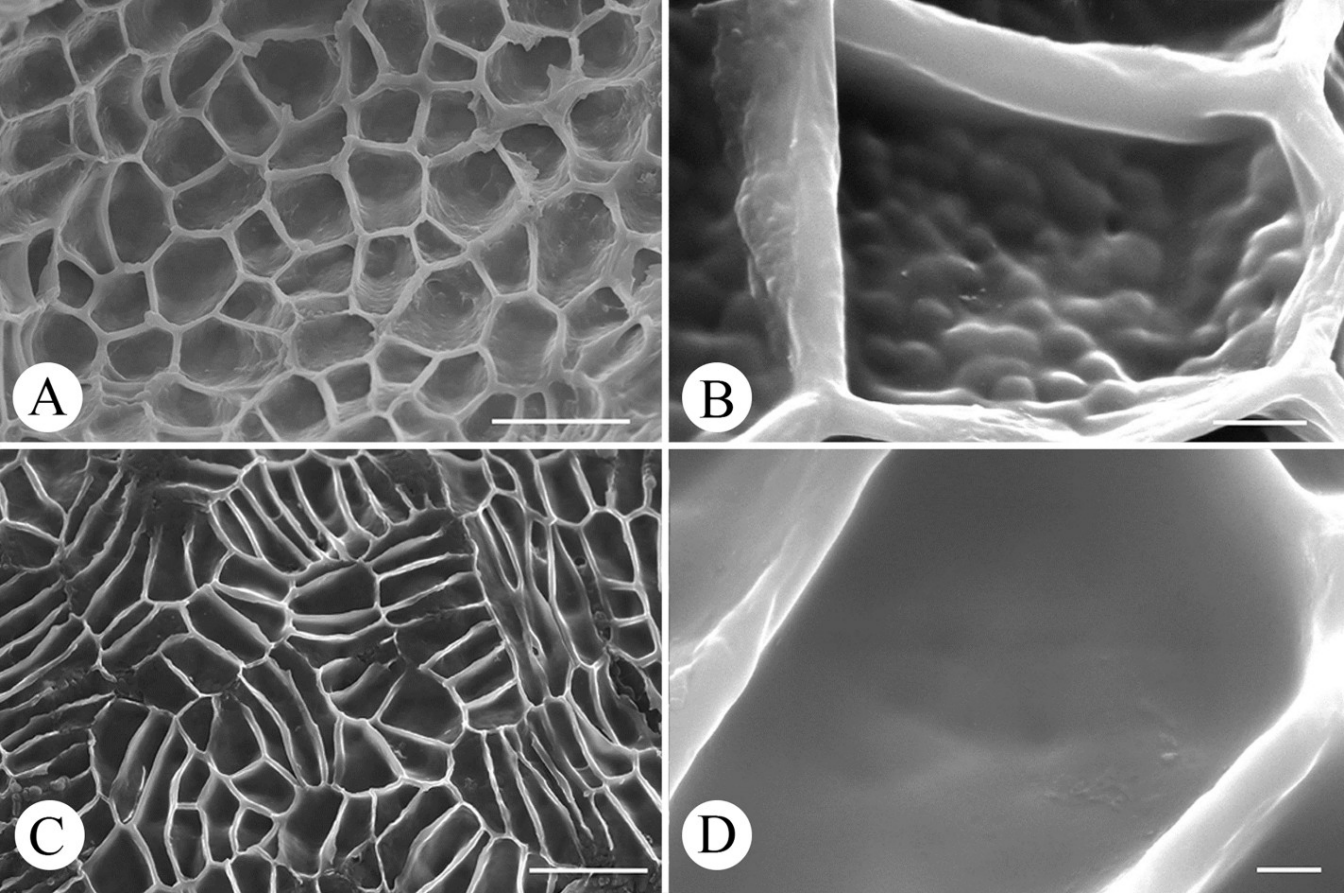
Epidermal cell morphology of sclerotesta of *Manglietia pubipedunculata* and *M*. *kwangtungensis* under scanning electron microscope. A, B, *M*. *pubipedunculata*; C, D, *M*. *kwangtungensis*; A, C, cell pattern and shape of sclerotesta surface; B, D, secondary cell wall sculpture of sclerotesta surface. Scale bar: A, C = 50 μm; B = 5 μm; D = 2 μm.

The two species had quite different characters under SEM, even though they had the same type of anticlinal cell wall boundary, which was convex linear. For *M*. *pubipedunculata*, the cell pattern on the surface of sclerotesta was alveolate consisting of pentagon or hexagon cells (Fig 6A and 6B), the average cell size was 33.5 × 14.7 μm, the thickness of outer periclinal cell wall was 3.2 μm, and there were papillate sculpture on secondary periclinal cell wall (Fig 6B). For *M*. *kwangtungensis*, the cell pattern on the surface of sclerotesta was mainly reticulate consisting of rectangular cells (Fig 6C and 6D), the average cell size was 35.7 × 12.8 μm, the thickness of outer periclinal cell wall was 2.2 μm, and secondary periclinal cell wall was smooth (Fig 6D).

### Species recognition

*Manglietia pubipedunculata* is similar to *M*. *kwangtungensis*, but it differs mainly in its appressed (vs. cocked and curly) pubescence (Fig 3C and 3I), shorter and thicker peduncles (5.5–7.5 × 0.7–0.8 cm vs. 7.2–12 × 0.6 cm) (Fig 2D and 2E), larger gynoecia (2.6–3.2 × 1.9–2.2 cm vs. 1.5–2.3 × 1.2–1.4 cm) with longer styles (7–8 mm vs. 4 mm) (Fig 2G and 2I), basal carpels (sparsely brown appressed pubescent vs. glabrous), less carpels (ca. 39 vs. 50–55) while more ovules per carpel (10–13 vs. 4–8). A more detailed macromorphological comparison is given in Table 1. Moreover, we observed the significant differences on micromorphology of their pubescence, thickness of leaves and sclerotesta. *M*. *pubipedunculata* has unicellular or bicellular pubescence (Fig 3B and 3C) with length of 364.2 μm, while *M*. *kwangtungensis* has tricellular pubescence (Fig 3H and 3I) with the length of 813.3 μm. *M*. *pubipedunculata* has aveolate cell pattern (Fig 6A) consisting of pentagon or hexagon cells (Fig 6A) with papilla on the secondary periclinal cell wall (Fig 6B), while *M*. *kwangtungensis* has reticulate cell pattern (Fig 6C) mainly consisting of rectangular cells (Fig 6C) with smooth secondary periclinal cell wall (Fig 6D). Our results based on macromorphology and micromorphology support *M*. *pubipedunculata* as a distinct species.

**Table 1:** Macromorphological comparison between *Manglietia pubipedunculata* and *M*. *kwangtungensis*.

Characters	*M*. *pubipedunculata*	*M*. *kwangtungensis*
Leaves	20–35×6.5–9.5 cm, with appressed pubescence beneath	12–25×4–8.8 cm, with cocked and curly pubescence beneath
Flowers	white, purplish-red inside of the base; outer 3, 8.8–9.8×3.8–4.8 cm; mid 3, 8.2–9.2×3.2–3.8 cm; inner 4–5, 7.2–7.5×2.5–3 cm	white; outer 3,5.5–7.5×2.8–3.5 cm; mid 3, 6.5–7×2.7–4 cm; inner 3–5, 4.8–6.5×2–2.5 cm
Peduncles	5.5–7.5×0.7–0.8 cm	6–12×0.6 cm
Stamens	1.6–1.7 cm	1.1–1.3 cm
Gynoecia	2.6–3.2×1.9–2.2 cm	1.5–2.3×1.2–1.4 cm
Styles	7–8 mm	3–4 mm
Carpels	ca. 39, sparsely brown appressed pubescence at basal carpels	50–55, glabrous
Ovules	(9)10-13(14)	(3)4-8(9)
Follicle apexes	3–5 mm	2–3 mm

### Phylogenetic position

**Fig 7:**
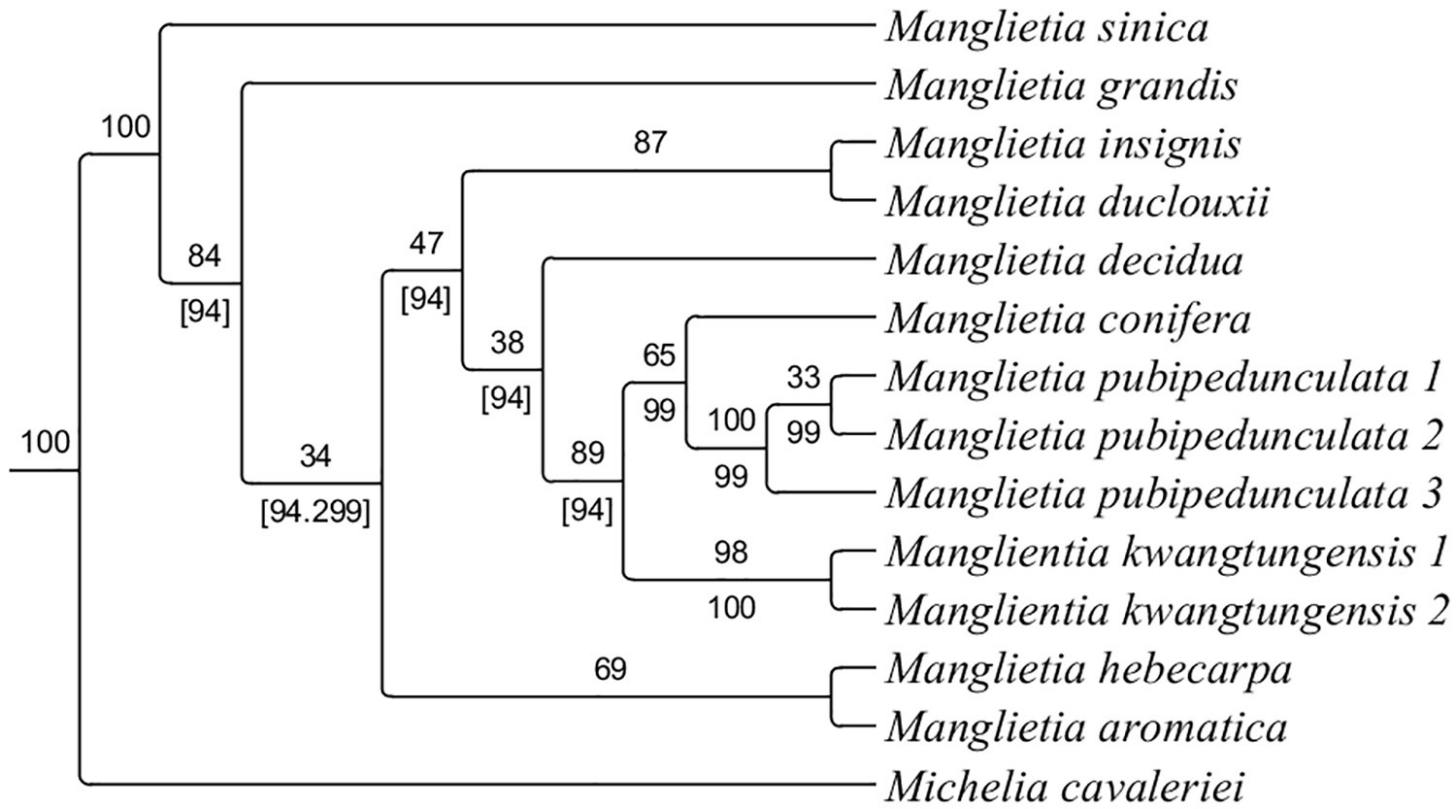
The ML consensus tree of *Manglietia* based on three molecular markers (PHYA, LEAFY, and *trn*H-*psb*A). Note: The topolopy of the MP tree is congruent with the ML tree. Numbers above and below the branches are ML maximum parsimony bootstrap values (BS) and MP maximum parsimony bootstrap values (BS), respectively. [] shows the tree of ML and MP conflicts in the node.

The aligned matrix contained 580 characters in the LEAFY dataset and 743 in the PHYA dataset. The aligned *trn*H-*psb*A matrix consisted of 373 characters. Based on the combined dataset of two nrDNA sequences (LEAFY and PHYA) and cpDNA sequence (*trn*H-*psb*A), MP and ML analyses yielded congruent topologies which is shown in Fig 7. The new species, *M*. *kwangtungensis* and *M*. *conifera* form a clade (ML bootstrap, BS = 89%, MP bootstrap, BS = 94%). Obviously, the three samples of *M*. *pubipedunculata* form a highly supported clade (ML bootstrap, BS = 100%, MP bootstrap, BS = 99%), and it is sister to *M*. *conifera*, but with weak support in ML analyses (ML bootstrap, BS = 65%, MP bootstrap, BS = 99%).

## Discussion

In our phylogenetic study, the new species, *M*. *conifera* and *M*. *kwangtungensis* form a clade, which shows the three species have relatively close relationship, which is consistent with morphological characteristics for having pendulous peduncles in the genus [43]. Compared to the glabrous peduncles and bracts of *M*. *conifera*, the new species *M*. *pubipedunculata* is more similar to *M*. *kwangtungensis* for having dense pubescence on peduncles and bracts, this is why we use these two species to make morphological comparative study.

Studies on leaf morphology were very important for the specimens examination because lots of specimens had no flowers and fruits [55]. Previous studies on the Magnoliaceae [18–20, 45, 56–61] showed the leaf characters have the taxonomic significance. Leaves may affect by the environment, but the affection just happened on the quantitative characters, the qualitative characters kept relatively stable [62, 63]. In our study, both the quantitative characters including the density and length of the pubescence, the leaf thickness, and the qualitative characters such as the hair type, are remarkably different.

For seed plants, the seed characters are of hereditary stability [64]. Researches demonstrated the type of chalazal region, the colour, shape, size, sculpture of surface and epidermal cell of sclerotesta had the taxonomic significance in the family, genera, and species [26, 27, 46, 64–66]. In the present study, notable differences were definitely found between *M*. *pubipedunculata* and *M*. *kwangtungensis*.

## Supporting information

S1 Text Specimens of its related species *Manglietia kwangtungensis* examined. (PDF)

S1 Table GenBank accession numbers for all the DNA sequences and voucher information. (XLS)

S2 Table Primers of PHYA, LEAFY and *trn*H-*psb*A used for amplification and sequencing in this study. (XLS)

S3 Table Data of leaf epidermis. (XLS)

S4 Table Data of leaf structure. (XLS)

S5 Table Data of seeds removing the exotesta and mesotesta. (XLS)

S6 Table Data of sclerotesta epidermal cell. (XLS)
