# Supplementary material for: Manglietia pubipedunculata (Magnoliaceae), a new species from Yunnan, China
Source: PLoS One. 2019 Mar 13;14(3):e0210254. doi: 10.1371/journal.pone.0210254 (PMC6415859; doi:10.1371/journal.pone.0210254)
Supplement: S1 Text — (PDF) [file pone.0210254.s001.pdf]

S1 Text. Specimens of its related species *Manglietia kwangtungensis* examined

Specimens collected from CHINA. Guangdong. Yingde: *Canton Christian College12179,12344* (IBSC & SYS, Isotypes), *P. Zeng 022* (SYS), *B. H. Liang 84463* (PE), *B. H. Liang 83574*, *84074*, *84463* (IBSC), *L. Deng 894* (IBSC & KUN), *X. Z. Wang 7353* (IBSC), *W. D. Xu 8556* (IBSC), *T. Hai 695* (IBSC & SYS), *X. P. Gao 50451* (IBSC), *Q. W. Zeng & X. M. Hu 00196*, *00197*, *00198* (IBSC); Lechang: *S. Q. Chen 3216* (PE, KUN, IBSC), *H. Y. Chen 10681* (KUN), *C. L. Tso 20508*, *20905*, *21173* (IBSC & PE), *21098* (IBSC & SYS), *B. Y. Chen 2478* (IBSC), *B. L. Deng 20153* (IBSC), *N. Q. Chen 42968* (PE & IBSC), *Y. Li 10681* (IBSC); Fengchuan: *C. Huang 164020* (PE, KUN, IBSC), *G. Q. Ding*, *L. Yu 6263* (IBSC), *G. L. Shi 14833* (IBSC), *J. Q. Zhang 83279* (IBSC), *B. L. Chen 87G-161* (SYS); Longmen: *G. C. Zhang 410* (IBSC), *Q. W. Zeng & X. M. Hu 00184*, *00185*, *00186*, *00253*, *00254* (IBSC); Lianshan: *P. X. Tan 58521* (PE, KUN, IBSC); Liannan: *P. X. Tan 59026* (PE, KUN, IBSC), *Z. S. Zhu 660* (IBSC); Huaxian: *C. Huang 164659* (PE, KUN, IBSC); Lianxian: *S. S. Lai & D. F. Huang 31* (GF); Conghua: *S. Q. Chen 17354* (IBSC), *Q. W. Zeng & X. M. Hu 007* (IBSC); Ruyuan: *N. H. Xia & N. Liu 335* (IBSC), *X. P. Gao 53514* (PE & IBSC), *Y. G. Liu 00417* (PE & IBSC); Dinghu: *G. L. Shi 11955*, *14301*, *12781* (IBSC); Huaiji: *B. H. Chen 1257* (IBSC), *S. C. Ng 3023* (PE); Deqing: *Y. G. Liu 01020* (GF & IBSC); Gaoyao: *G. Q. Ding & G. L. Shi 10269* (IBSC), *G. L. Shi & S. D. Huang 2362* (IBSC); Yunfu: *Z. Huang 37283* (PE & IBSC); Lianyang: *Y. Q. Cheng 170279* (IBSC); Renhua: *L. Deng 7600* (PE); Yangchun: *H. G. Ye & N. Liu 517* (IBSC), *W. B. Liao & Z. Y. Su 0169* (SYS); Yunan: *H. G. Ye & N. Liu 2751* (IBSC); Huanjiang: *C. F. Pang 34* (IBSC); Places unknown: *X. L. Zou & B. L. Deng 20248* (IBSC), *H. Y. Chen 5860* (IBSC), *S. Z. Xin 9966* (IBSC), *B. L. Chen et al. 150, 370* (SYS), *B. S. Wang & H. X. Qiu 140* (SYS), *Y. C. Huang 84-017* (SYS), *B. L. Chen & C. X. Ye 80115* (SYS), *Q. Feng & P. Zeng 10838* (SYS).

Specimens collected from CHINA. Guangxi. Hexian: *H. C. Chen et al. 500183* (IBSC), *Z. T. Li 603835* (IBSC); Cangwu: *S. Q. Chen 10140* (PE & IBSC); Places unknown: *T. J. Zhang 9240*, *9241* (YCP), *unknown 500183* (SYS).

Specimens collected from CHINA. Hunan. Yizhang: *Q. Lin 175* (IBSC), *L. H. Liu 010685* (IBSC); Zixing: *H. S. Liao 15418* (IBSC); Mangshan: *M. X. Huang 112789* (IBSC), *G. Z. He 4678* (IBSC); Places unknown: *R. Z. Zhou 801307* (IBSC), *Z. C. Luo 1386* (PE), *H. S. Liao 7704-1-118* (PE).
